# Harnessing metamaterials for efficient wireless power transfer for implantable medical devices

**DOI:** 10.1186/s42234-023-00136-z

**Published:** 2024-03-06

**Authors:** Sultan Mahmud, Ali Nezaratizadeh, Alfredo Bayu Satriya, Yong-Kyu Yoon, John S. Ho, Adam Khalifa

**Affiliations:** 1 Department of Electrical and Computer Engineering, University of Florida, Gainesville, FL 32611 USA; 2 Department of Electrical and Computer Engineering, National University of Singapore, Singapore, Singapore

**Keywords:** Wireless power transfer, Metamaterials, Implantable medical devices, Miniaturized

## Abstract

Wireless power transfer (WPT) within the human body can enable long-lasting medical devices but poses notable challenges, including absorption by biological tissues and weak coupling between the transmitter (Tx) and receiver (Rx). In pursuit of more robust and efficient wireless power, various innovative strategies have emerged to optimize power transfer efficiency (PTE). One such groundbreaking approach stems from the incorporation of metamaterials, which have shown the potential to enhance the capabilities of conventional WPT systems. In this review, we delve into recent studies focusing on WPT systems that leverage metamaterials to achieve increased efficiency for implantable medical devices (IMDs) in the electromagnetic paradigm. Alongside a comparative analysis, we also outline current challenges and envision potential avenues for future advancements.

## Introduction

Essential to contemporary healthcare, IMDs offer invaluable support in disease management and preventative care. Their applications span from established technologies like cardiac pacemakers and drug delivery systems to emerging innovations such as closed-loop neural stimulators and glucose-monitoring implants. While IMDs have revolutionized healthcare, their adoption is hindered by several technological limitations. Most notably, the invasiveness of the surgery required to implant these devices is a significant barrier to broader usage. This is often due to the bulky battery that current devices contain, which requires subsequent replacement surgeries to operate beyond their useful lifespan. A promising avenue to address the limitations of batteries is through wireless power transfer, enabling rechargeable or battery-free IMDs that can function indefinitely within the body. WPT enables miniaturized devices that can be implanted through minimally invasive procedures and the fabrication of novel types of devices that are impractical with wired power delivery (Khalifa et al. 2019; Shadid and Noghanian 2018; Khalifa et al. 2021).

WPT systems are designed with the primary goal of maximizing PTE. This is influenced by multiple parameters, such as the spatial relationship and alignment between the transmitter and receiver and the system's operating frequency. Optimal design must synergistically address these factors to achieve peak PTE (Khalifa et al. 2021). Concurrently, mitigating the Specific Absorption Rate (SAR), a metric that quantifies the electromagnetic energy absorbed by biological tissue, is a critical challenge. The Food and Drug Administration imposes caps on allowable SAR levels, effectively constraining the transmitter power (Fields 1997).

Various strategies have been explored to surmount these challenges. One notable method employs multi-objective optimization algorithms to maximize PTE for specific transmitter and receiver geometries (Khalifa et al. 2023; Xu et al. 2022). One method to increase the PTE is to use an array of coils or antennas. This method requires more space, but it can enhance the PTE by increasing the coupling factor between the array of transmitters and the receiver (Das et al. 2021; Nasrollahpour et al. 2021; Zaeimbashi et al. 2021). Electric field or capacitive coupling is another method where better efficiency is achievable with a lower distance between the transmitter and receiver coil (Jegadeesan et al. 2017). Hybrid region coupling is also addressed where maximum gain can be achievable with broad bandwidth (Wen et al. 2023). An increasingly compelling strategy involves the use of metamaterials – engineered materials with unique electromagnetic properties not found in nature. The deployment of metamaterials offers a promising avenue for significantly boosting PTE across both near-field and far-field electromagnetic domains (Li et al. 2021; Adepoju et al. 2022; Lee and Yoon 2020a; Song et al. 2021; Wang et al. 2011; Lipworth et al. 2014; Che et al. 2014; Sun et al. 2018; Rong et al. 2021; Zhou et al. 2022).

**Fig. 1:**
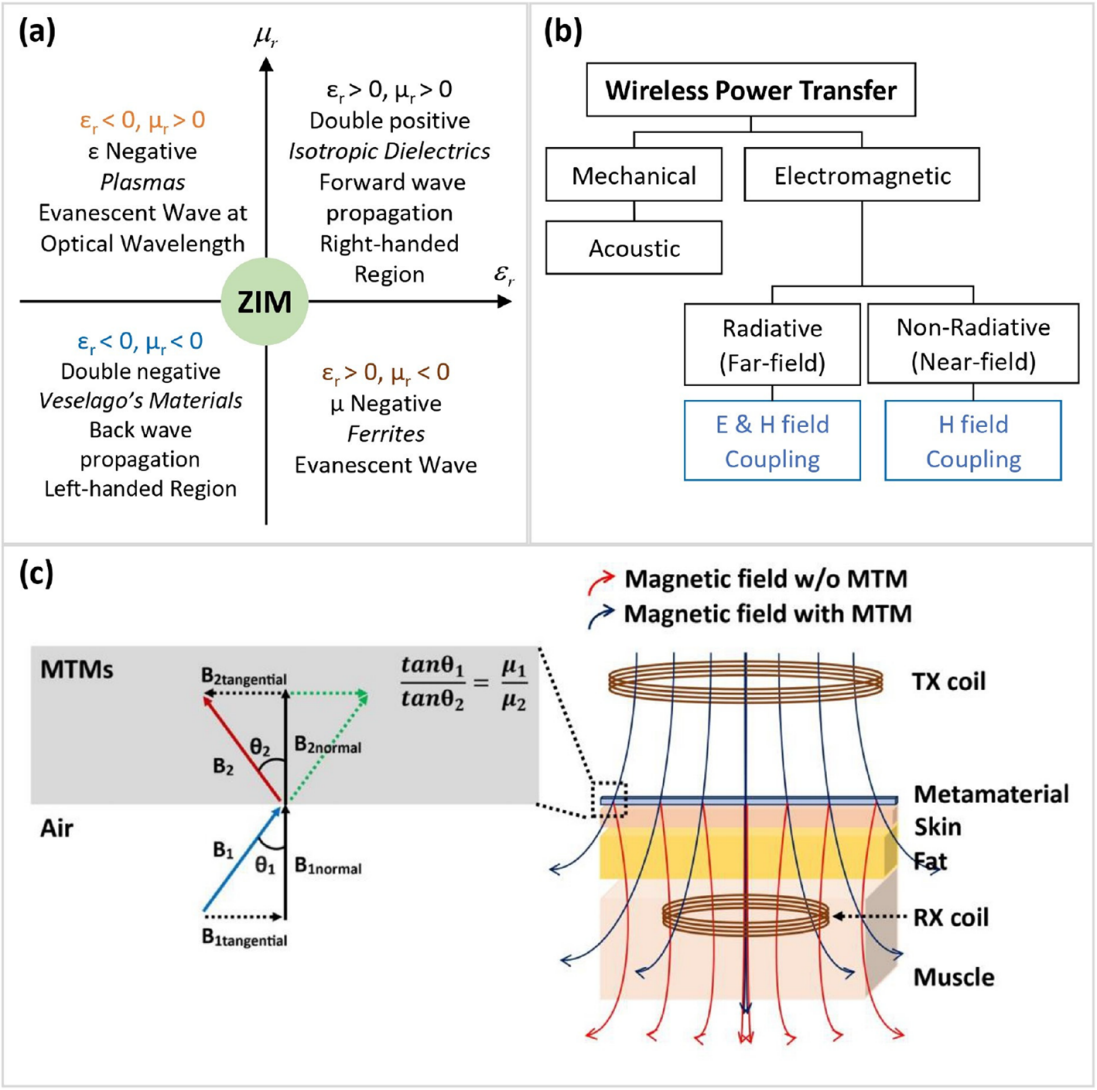
**a** Classification of conventional material and four types of metamaterials in four different regions (ZIM: Zero Index Metamaterials), (**b**) Classification of common WPT methodologies which mostly focused on metamaterials, (**c**) Representation of a bio-implant WPT system including metamaterials where the electromagnetic wave is guiding the metamaterial for better PTE

Metamaterials are notable for their applications across various fields, such as biomedical systems, sensing, antennas, lenses, cloaking, and energy harvesting (Hussain et al. 2023; Ho and Li 2022; Lee et al. 2022; Alam et al. 2021; Haxha et al. 2018; Islam et al. 2015; Rana et al. 2023; Hossain et al. 2022). Figure 1a categorizes metamaterials into four primary types: double negative, epsilon negative, mu negative, and zero-index metamaterials. One dramatic example of their distinctive properties is negative permittivity and permeability, leading to negative refraction. This property enhances evanescent waves at deep sub-wavelength scales, increasing PTE through resonant coupling. Additionally, the magnetic field originating from the transmitter coil undergoes two distinct directional shifts as it passes through the metamaterial and reaches the receiver coil. The first shift occurs at the interface between the transmitter's air or PCB layer and the metamaterial, while the second occurs at the interface between the metamaterial and the skin. These directional modifications serve to extend the transfer distance and facilitate the generation of an evanescent wave (Chen et al. 2017).

Various classifications of prevalent WPT techniques for biomedical applications are depicted in Fig. 1b. These techniques range from mechanical means, such as acoustic methods (Basaeri et al. 2016), to electromagnetic approaches, including radiative and non-radiative techniques (Ahire and Gond 2017; Kim et al. 2017). Notably, metamaterials are utilized within the realm of electromagnetic WPT approaches. As depicted in Fig. 1c, positioning a metamaterial between the transmitter and the receiver can effectively concentrate power onto the IMD. Notably, most metamaterials research focuses on the transmitter, primarily because it often resides outside the human body, thus allowing for greater design flexibility.

While WPT systems utilizing metamaterials have recently garnered attention across diverse applications (Lee and Yoon 2022; Jiang et al. 2021; Cheng et al. 2016; Kim and Seo 2014), their integration into biomedical contexts remains comparatively underexplored. This review aims to bridge this gap by presenting recent advancements in WPT systems that employ metamaterials specifically for IMDs as well as discussing the challenges and limitations followed by future trends.

## Wireless power transfer with metamaterials for implantable devices

**Fig. 2:**
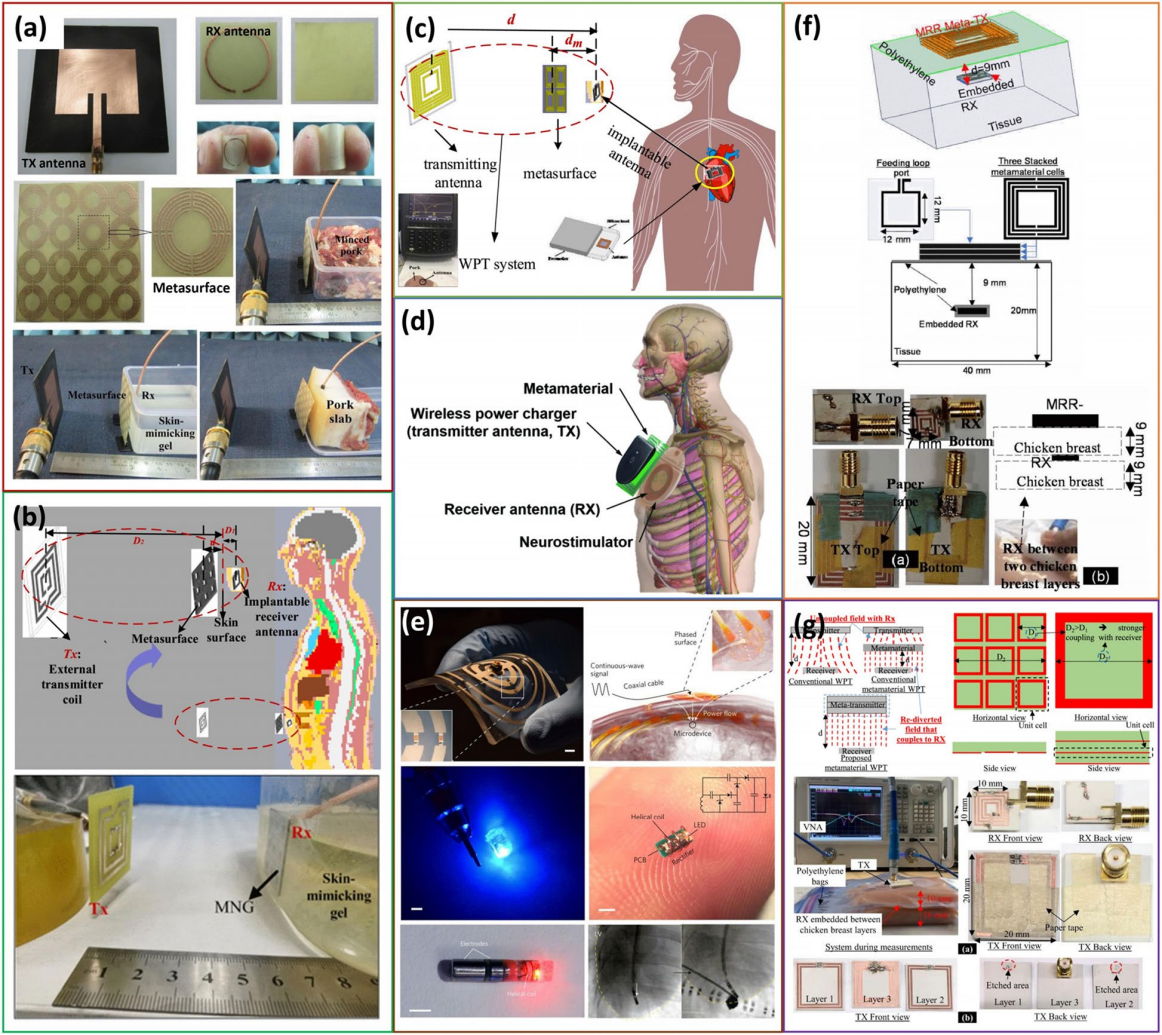
Representation of several recent state-of-the-art literature related to WPT systems with metamaterial. **a** (Shaw and Mitra 2019), (**b**) reprinted with permission, copyright IEEE (Li et al. 2018), (**c**) reprinted with permission, copyright IEEE (Wang et al. 2021), (**d**) reprinted with permission, copyright IEEE (Chen et al. 2019), (**e**) Adapted from (Agrawal et al. 2017), (**f**) reprinted with permission, copyright IEEE (Alshhawy et al. 2022), and (**g**) Adapted from (Pokharel et al. 2021)

Metamaterials boost the PTE by strategically placing it between the transmitter and receiver or functioning as the transmitter itself. Recently, a few groups have relied on metamaterials to boost the PTE and increase the distance between the Tx and Rx, as demonstrated in Fig. 2. All of them can be useful for bio-implant application, which is the main focus of this review. For a detailed overview, Table 1 provides a comprehensive summary of each reviewed paper, including a figure of merit (FOM) for each. This FOM evaluates the performance of individual WPT systems, considering efficiency (η), transmission distance (d), and receiver coil area (A) as determined by the equation depicted below (Zargham and Gulak 2015),

**Table 1:** Comparative overview of recent metamaterials demonstrated to enhance powering of IMDs

	**Metamaterials situated between transmitter and receiver coil**				**Metamaterial acting as transmitter coil**		
Reference	(Shaw and Mitra 2019)	(Li et al. 2018)	(Wang et al. 2021)	(Chen et al. 2019)	(Agrawal et al. 2017)	(Alshhawy et al. 2022)	(Pokharel et al. 2021)
**Metamaterial Properties**							
** Total Size (mm**^**2**^**)**	30 × 30	21 × 21	60 × 60	500 × 500	60 × 60	20 × 20	60 × 60
** Operating Frequency (MHz)**	430	272–1504	1600	13.56	1600	50	50
**Transmitter Size (mm**^**2**^**)**	90 × 90	40 × 40	N/A	N/A	N/A	N/A	N/A
**Receiver Size (mm**^**2**^**)**	12 × 12	10 × 12	12 × 12	10 × 10	1.5 × 3	7 × 7	10 × 10
**Tx to Metamaterial Distance (mm)**	53	70	50	5	N/A	N/A	N/A
**Rx to Metamaterial Distance (mm)**	18	13	6	5	42	9	10
**PTE without metamaterial (%)**	0.34	0.002	0.01	N/A	N/A	N/A	N/A
**PTE Improvement with Metamaterial (%)**	1.11	1.6	1.45	5	N/A	2.5	6
**FOM**	4.89	2.68	0.18	0.63	4.37	5.31	6.00
**Max Excitation Power in Tx coil (mW)**	180	N/A	N/A	N/A	800	130	168

$$FOM=\frac{\eta \cdot {d}^{3}}{{A}^{1.5}}$$

### Metamaterials situated between transmitter and receiver coil

#### Enlarging the effective aperture by high refractive index CSSRR metasurface

Shaw et al. proposed a novel approach to increase the near-field WPT efficiency using a high-refractive-index circular spiral split ring resonator (CSSRR) metasurface for IMDs operating in the 2.40–2.48 GHz band (Fig. 2a) (Shaw and Mitra 2019). A metasurface is a type of metamaterial that has two dimensions. In their study, the researchers employed a microstrip patch antenna as the transmitting (Tx) coil and a planar loop as the receiving (Rx) coil. They demonstrated improved Power Transfer Efficiency (PTE) through simulation and measurement. A primary factor contributing to this enhancement is the increased effective aperture of the Rx coil. A flexible substrate was used to address the rigidity of the skin. The authors also investigated the effects of different skin layer thicknesses and misalignments at a distance of 50 mm. This type of IMD has potential applications as a neurostimulator, pressure sensor, and glucose sensor.

#### Increasing the induced current by dual-layer square spiral MNG metasurface

Li et al. introduced an advanced WPT system, augmented with a mu-negative metasurface (MNG), tailored specifically for biological applications (Fig. 2b) (Li et al. 2018). This miniaturized wearable device is positioned directly on the human skin and incorporates a negative permeability metasurface, optimally enhancing the PTE. The team embeds a dual-band receiver just 3 mm beneath the skin, capable of simultaneous power transmission through the lower band (402 MHz to 405 MHz) and data transfer via the higher band (1.6 GHz and 2.4 GHz). To ensure seamless wireless power connectivity, they utilize a conformal, strongly-coupled magnetic resonator coil as the transmitter. The dual-layer induced current increased the metasurface, which was transmitted to the receiver side.

#### Improving magnetic resonant behavior by MNG metasurface

Wang et al. introduce a new concept by integrating a bandwidth-enhanced miniaturized antenna within the silicone header of a cardiac pacemaker tailored for WPT applications (Fig. 2c) (Wang et al. 2021). This antenna achieves an impressive S_11_ across a broad frequency span (from 272 to 1504 MHz). Central to its design is the strategic placement of an MNG metasurface array between the Tx and Rx coils, a significant advancement for systems that might experience misalignment between these coils. Integration of the metasurface array significantly bolsters the PTE and effective transmission distance even with the human body’s inherent absorption capabilities. The measured and simulated results agree well, confirming the suitability of the proposed antenna for WPT systems for IMDs.

#### Amplitude enhancement of EM wave by SRR metamaterial

Chen et al. developed a 13.56 MHz metamaterial specifically tailored for powering IMDs (Fig. 2d) (Chen et al. 2019). The design of each metamaterial unit incorporates two copper wire layers, each coiled in three clockwise turns. The cumulative effect of a 5 × 5 array of these metamaterials augments the coupling of evanescent waves. The research further integrates an LC resonant tank, a critical addition to counteract efficiency losses from the eddy currents introduced by the device packaging and any possible misalignment. These innovative aspects enhance the system's PTE, making it exceptionally compatible with conventional coils. After adding the metamaterial array, the coil achieves an efficiency boost from 46 to 51%.

### Metamaterials acting as transmitting coil

#### Improving magnetic field by conformal phased surface metamaterial

Agrawal et al. (Agrawal et al. 2017) proposed a metasurface-based conformal phased surface transmitter to power deep IMDs wirelessly in the mid-field regime (Fig. 2e). This approach demonstrated a flexible/stretchable electromagnetic structure using subwavelength resonant elements to enhance the wireless power transfer efficiency and distance, in contrast to traditional methods due to the conversion of evanescent and propagating fields at the air-tissue interface. Another vital factor for the high-power transfer is the removal of loading elements on the surface, which eliminates the focal spot. A 6 × 6 cm^2^ conformal Tx coil transfers 0.83 mW over 4 cm distance on saline and 0.45 mW over 4.2 cm thick porcine tissue with a 1.5 mm diameter helical Rx coil. The conformal shape also increases the coupling on the curved body.

#### Low magnetic loss stacked MRR metamaterial

Alshhawy et al. introduce a WPT system for IMDs that utilizes a multi-ring resonator (MRR) metamaterial characterized by its low magnetic loss (Fig. 2f) (Alshhawy et al. 2022). This MRR metamaterial, arranged in a stacked formation, functions as the WPT transmitter, targeting a compact receiver embedded within biomedical tissue to augment PTE with initial Tx-Rx distance. To achieve this, the researchers implemented a metamaterial-based Tx coil, called meta-TX, which corresponded to a considerably smaller receiver coil within the IMD. Their prototype, when tested, exhibited a promising efficiency of 51% at 50 MHz with a Tx-Rx distance of 9 mm.

#### Decreasing the resonance change by stacked SRR metamaterials

To address the limitations of conventional resonant inductive coupling WPT systems, Pokharel et al. proposed a novel coaxially aligned geometry based on metamaterials with near-zero permeability properties (Fig. 2g) (Pokharel et al. 2021). The geometry consists of a stacked split ring resonator metamaterial driven by a feeding inductive loop, which acts as a WPT transmitter for an implanted receiver inside the tissue. They demonstrate that the proposed metamaterial-based WPT system has superior misalignment performance due to the decrement of the resonant change, and their transmission is insensitive to tissue characteristics.

## Comparative analysis

Table 1 demonstrates various methodologies, parameters, and performance metrics from recent peer-reviewed articles focused on WPT for biomedical implants. The table underscores the absence of a one-size-fits-all solution, highlighting the complex trade-offs among efficiency, transmission range, device dimensions, safety protocols, and biocompatibility instead. PTE increment mostly depends on the manipulation of EM waves in such a way that the Rx coil receives the highest power sent from the Tx coil. From the previous section, it can be seen that different groups followed different ways to improve the PTE. Considering (Shaw and Mitra 2019; Li et al. 2018; Chen et al. 2019), reference (Wang et al. 2021) will not be an option as it uses a dual-layer metasurface with lower FOM. Besides, FOM for (Shaw and Mitra 2019) is higher than other work as it enlarges the effective aperture by a high refractive index metasurface. In addition, separating the metamaterial from the Tx provides greater resilience to the WPT system, as the Tx position remains adjustable. On the contrary, such a WPT system needs a relatively larger space. On the other hand, it can be noticed that when metamaterials are acting as the Tx coil, there's a reduction in system size alongside improved efficiency. Even though (Pokharel et al. 2021) provided better FOM than (Agrawal et al. 2017; Alshhawy et al. 2022), due to the conformal phased surface (Agrawal et al. 2017) can be a good option for real-life application while using flexibility as a tool for PTE improvement. In addition, (Alshhawy et al. 2022; Pokharel et al. 2021) are promising as they reduced the sensitivity to tissue characteristics with a metamaterial transmitter coil. It can be examined from the literature that metamaterials-based WPT systems for the IMDs are mostly radiative structures whereas other examples with non-radiative structures are also available. All referenced works adhered to the SAR limit, as mandated by regulations. In summary, metamaterials emerge as a critical component in numerous methodologies, conferring multiple advantages such as enhanced PTE, miniaturized system dimensions, and a reduction in sensitivity to the variable properties of biological tissues.

## Challenges and limitations of using metamaterials powering IMDs

While WPT augmented by metamaterials holds considerable promise for enhancing power transmission efficiency to biomedical implants, it is not without its challenges and limitations. Extensive research into this area has surfaced a host of issues that complicate the straightforward application of this innovative technology. In this section, we outline some of the challenges and potential solutions.

### Frequency and size

One of the inherent challenges lies in the delicate balance between the frequency and size of metamaterials. Lowering the operating frequency is desirable to lower the SAR. However, the working frequency of metamaterials is inversely proportional to their dimensions. Consequently, a reduction in frequency necessitates an increase in the size of the metamaterial. In some instances, this scaling can result in metamaterial dimensions exceeding those of the transmitter coil, rendering the setup impractical for certain applications. However, metamaterial can work in subwavelength which can resolve the problem (Agrawal et al. 2017). Another solution to this challenge is to change the resonator shape and increase the number of material options, which can lead to better PTE at lower frequencies (Shan et al. 2022; Wang et al. 2011).

### User-friendliness

While an optimal distance exists between the metamaterial and the transmitter for achieving peak performance, user-friendliness imposes constraints on this distance. For example, strapping a large portable casing to one's head to power brain implants is impractical. Such a setup would be uncomfortable and cumbersome and significantly hinder routine activities. Unfortunately, decreasing the distance between the Tx and the metamaterials introduces a metallic component near the Tx coil which can complicate the design process and may inadvertently alter the transmitter's electromagnetic properties. This problem can be addressed by changing the resonator structure or materials of the metamaterials (Islam et al. 2017; Zhou et al. 2008). Designing tunable metamaterial structures can also be a robust solution to this problem (Lee and Yoon 2020b; Boardman et al. 2011). In addition, while considering the IMDs, conformal metasurface can significantly improve the user-friendliness (Tian et al. 2023; Hajiaghajani et al. 2022).

### The angle of the incident wave

Another critical consideration is maintaining consistent transmission efficiency and resonance frequency irrespective of the angle of the incident wave. Defined as the angle between the incoming electromagnetic wave and the metamaterial surface's normal, the angle of the incident wave can substantially influence the metamaterial's effective refractive index and impedance. When the angle of the incident wave changes, the effective refractive index and impedance of the metamaterial may also change, resulting in reflection, refraction, or scattering of the wave. This can reduce the power transfer efficiency and shift the resonance frequency of the metamaterial. Therefore, it is desirable to design polarization-independent and angle-insensitive metamaterials, meaning that they can maintain their performance regardless of the orientation of the incident wave. One possible way to achieve this is to use symmetrical and uniform structures, such as bi-air-hole dielectric resonators or stacked split ring resonators. These structures can create destructive interference between superradiant and subradiant modes, leading to an electromagnetically induced transparency (EIT) effect. The EIT effect can enhance the transmission and dispersion of the metamaterial, making it suitable for slow light and sensing applications (Zhou et al. 2022; Pokharel et al. 2021; Zhu et al. 2018; Pokharel et al. 2021).

### Design process

The design process for metamaterials and metasurfaces is still in a nascent stage, and standardized procedures are lacking. In addition, the resonator layer plays a very important role in designing metamaterials. Controlled resonator layer design and materials choice are the main keys to this challenge. However, researchers are nowadays proposing methods such as deep learning and pixelated methods to design metamaterials (Rana et al. 2023; Bessa et al. 2019; Bui et al. 2020; Ghaderi et al. 2018). Moreover, tunable metamaterials or Huygens metasurfaces can resolve this issue (Lee and Yoon 2020b; Ataloglou et al. 2021; Epstein and Eleftheriades 2014).

## Future trends and conclusion

Although substantial progress has been made in the utilization of metamaterials for WPT in biomedical applications, various challenges remain unaddressed. Metamaterials offer the unique advantage of having multiple ‘zones’ with differing electromagnetic properties, functioning akin to lenses. Exploiting this multi-region architecture allows for greater manipulation of electromagnetic fields, offering potential improvements in WPT systems. This area is largely unexplored, so it emerges as a promising frontier for further investigation. Furthermore, the utilization of metamaterials in mid- and far-field WPT systems has yet to be rigorously investigated, particularly for IMDs. As such, enriching mid- or far-field WPT through the incorporation of metamaterials represents a promising avenue for further research.

The miniaturization of the system is one of the main challenges facing the integration of metamaterials into WPT. Given that the properties of metamaterials depend on the resonator structure, modifications to the resonator layer can produce more efficient unit cells for WPT. It has been previously documented that the scattering parameter of metamaterials experiences significant fluctuations in response to changes in the shape of the resonator layer, variations in the thickness of the dielectric layer, or shifts in the constituent materials. Consequently, it can be asserted that the feasibility of system miniaturization is a distinct possibility with the incorporation of metamaterials. Furthermore, flexible or stretchable metamaterial structures as Tx coils or media between Tx and Rx coils, with features such as broadband operation and polarization insensitivity, are promising future directions. However, the key use cases of metamaterial-based WPT for IMDs need to be identified before further development can proceed, such as, frequency and size limitations of metamaterials and real-life application. Moreover, the proper design process of metamaterial is a challenge while working with IMD’s, considering the high permittivity of human tissue.

In summary, adding metamaterials in IMD’s is a new and promising field with the potential advantages of higher PTE, more extended range, and miniaturization. However, while metamaterials have shown great promise, a few challenges must be addressed before they can be widely applied in real-world applications. Future research in this area could lead to innovative solutions that have the potential to revolutionize healthcare diagnostics.

## Data Availability

Not applicable.

## Abbreviations

WPT: Wireless Power Transfer
TX Coil: Transmitter Coil
RX Coil: Receiver Coil
PTE: Power Transfer Efficiency
IMD: Implantable Medical Devices
SAR: Specific Absorption Rate
FOM: Figure of Merit
